# Dysfunctional anxiety in pandemic: Relationship to infodemic and behavior

**DOI:** 10.1192/j.eurpsy.2021.780

**Published:** 2021-08-13

**Authors:** S. Kumchenko, E. Rasskazova, A. Tkhostov

**Affiliations:** 1 Faculty Of Psychology, Lomonosov Moscow State University, Moscow, Russian Federation; 2 Department Of Neuro- And Pathopsychology, Faculty Of Psychology, Lomonosov Moscow State University, Moscow, Russian Federation

**Keywords:** infodemic, Dysfunctional anxiety

## Abstract

**Introduction:**

Anxiety are among the most common (Huang, Zhao, 2020, Rajkumar, 2020, Roy et al., 2020) and stable (Wang et al., 2020) mental complaints in a pandemic situation. Based on cognitive approach (Beck, Emery, Greenberg, 2005) one should differentiate unrealistic (dysfunctional) anxiety as well as different types of anxiety (Roy et al., 2020).

**Objectives:**

The aim was to reveal relationship of different types of anxiety with the search for information about coronavirus and protective behavior.

**Methods:**

In April 2020 (2-3 weeks of self-isolation regimen) 409 respondents not infected by coronavirus (186 men, 223 women) aged 18 to 64 years appraised their anxiety of infection and pandemic consequences (Cronbach’s alphas .77-.82), the degree to which anxiety disturbs their usual activities, the frequency of tracking information about the coronavirus (.75) and various protective actions against the coronavirus (.76).

**Results:**

17.1% reported that anxiety disturbed their activities. Anxiety of pandemic negative consequences was more prominent than anxiety of infection and was unrelated to age and gender. Anxiety of infection was higher in females (t=-5.48, p<.01, η=.26) and elder people (r=.20, p<.01). Both anxiety of infection and of pandemic consequences was equally related to information tracking and protective behavior (r=.25-.36, p<.01). Dysfunctional anxiety was unrelated to adherence to self-isolation (r=.08) but was related to information tracking (r=.21, p<.01).

**Conclusions:**

Dysfunctional anxiety is unrelated to self-isolation and should be differentiated from realistic anxiety in studies of pandemic. Research is supported by the Russian Foundation for Basic Research, project No. 20-04-60072.

